# Public attitudes towards COVID‐19 contact tracing apps: A UK‐based focus group study

**DOI:** 10.1111/hex.13179

**Published:** 2021-01-12

**Authors:** Simon N. Williams, Christopher J. Armitage, Tova Tampe, Kimberly Dienes

**Affiliations:** ^1^ Centre for People and Organisation School of Management Swansea University Swansea UK; ^2^ Department of Medical Social Sciences Feinberg School of Medicine Northwestern University Chicago IL USA; ^3^ Manchester Centre for Health Psychology University of Manchester Manchester UK; ^4^ Manchester University NHS Foundation Trust Manchester Academic Health Science Centre Manchester UK; ^5^ World Health Organization Geneva Switzerland

**Keywords:** contact tracing, COVID‐19, public attitudes, public health

## Abstract

**Background:**

During the 2020 COVID‐19 pandemic, one of the key components of many countries’ strategies to reduce the spread of the virus is contact tracing.

**Objective:**

To explore public attitudes to a COVID‐19 contact tracing app in the United Kingdom.

**Setting:**

Online video‐conferencing.

**Participants:**

27 participants, UK residents aged 18 years and older.

**Methods:**

Qualitative study consisting of six focus groups carried out between 1st‐12th May, 2020 (39‐50 days into the UK ‘lockdown’).

**Results:**

Participants were divided as to whether or not they felt they would use the app. Analysis revealed five themes: (1) lack of information and misconceptions surrounding COVID‐19 contact tracing apps; (2) concerns over privacy; (3) concerns over stigma; (4)concerns over uptake; and (5) contact tracing as the ‘greater good’. Concerns over privacy, uptake and stigma were particularly significant amongst those stated they will not be using the app, and the view that the app is for the ‘greater good’ was particularly significant amongst those who stated they will be using the app. One of the most common misconceptions about the app was that it could allow users to specifically identify and map COVID‐19 cases amongst their contacts and in their vicinity.

**Conclusions:**

Our participants were torn over whether digital contact tracing is a good idea or not, and views were heavily influenced by moral reasoning.

**Patient or Public Contribution:**

No patients were involved in this study. The public were not involved in the development of the research questions, research design or outcome measures. A pilot focus group with participants not included in the present paper was used to help test and refine the focus group questions. Summary results were disseminated via email to participants prior to publication for feedback and comment.

## 1 BACKGROUND

As of September 16, 2020, COVID‐19 accounted for approximately one million deaths globally, approximately 40,000 of which were in the UK, with total numbers of cases and deaths increasing daily.^1^ The novelty of the SARS‐CoV‐2 virus means that, at present, no vaccines or antiviral drugs are available. In the absence of biomedical interventions to reduce morbidity and mortality from COVID‐19, non‐pharmaceutical interventions, such as physical distancing, hand hygiene and restricted movement, have been proposed, including the ‘lockdown’ policy of only leaving the home when essential (implemented in the UK on March 23, 2020).^2^ Although highly effective as a means to significantly slow the transmission of the virus (that is, to lower its reproductive (R0) value) in the population, lockdown is a strict strategy that is having number of significant adverse impacts, including wider economic impacts for society and social and psychological impacts for individuals.^3^, ^4^ As such, stringent lockdowns are necessarily a temporary and relatively short‐term measure.

One of the key components of many countries’ post‐lockdown strategies is the use of contact tracing. Contact tracing is where those in close contact with individuals, who report symptoms indicative of an infectious disease and/or test positive for the disease, are identified and given appropriate medical instruction. It has successfully been used to control the spread of other novel communicable diseases, such as Ebola.^5^ Traditionally, contact tracing relies on a team of health workers to track possible transmissions manually, through interviews with those who test positive for an infectious disease, in order to work out with whom they have recently been in close contact. Contacts deemed at risk are then informed of the fact they have been exposed and are then advised (or in some countries required) to self‐isolate. Several countries, most notably South Korea and Singapore, have successfully used contact tracing (primarily using manual tracing) methods at an early stage in the outbreak in order to dramatically slow the spread of, and reduce the total number of cases and deaths from, COVID‐19.^6^ However, while manual contact tracing has proven successful at early stages in the outbreak, where total number of cases are very low, it has been argued that doing so at a later stage, when total numbers of infections are much higher, is problematic.^7^ Firstly, manual contact tracing on a large scale is highly resource‐intensive. For instance, in the UK an initial 18‐25 000 contact tracers were employed during May 2020.^8^ Secondly, it has been argued that due to the speed at which COVID‐19 spreads, manual contact tracing might be too slow to be effective on its own.^9^ As such, digital contact tracing has been proposed and is being explored in a number of countries, including the UK, as a supplement to manual contact tracing.^10^

The UK’s path to providing a contact tracing app has been controversial and subject to much discussion and critique in the UK media.^11^ Essentially, contact tracing apps work by keeping a record of other smartphones they have been in close proximity with, via the phones’ built‐in Bluetooth signals that are anonymously swapped via a ‘digital handshake’. Initially, the UK government proposed a ‘centralized model’, where contact data are stored in a cental database and not exclusively on the phones themselves. On 12th April, the UK government announced the new centralized NHSX (the technology arm of the UK’s National health Service) contact tracing app, a pilot trial of which was launched on the Isle of Wight (a small island with a population of approximately 140 000) on 4th May.^12^ The centralized approach contrasted with the version developed by the major technology companies Apple and Google (a ‘decentralized approach’), where contact information is only stored on the phones’ handsets, a model being used in a number of other countries like Germany and Switzerland.^12^ On the 18th June, the UK government abandoned its app, in favour of the decentralized model, after releasing details of a number of problems identified in the pilot, including the fact that when used on Apple iPhones, only around 4% of contacts were successfully identified.^13^ The new, decentralized app was later officially released on September 24, 2020. As discussed below, focus groups for this study were conducted between 1st‐12th May 2020, prior to the app's eventual release, and at a time when the UK was proposing its NHSX centralized app would be used.

Modelling evidence suggests that in order for digital contact tracing to be effective, use of contact tracing apps would need to be very high, with 80% of smartphone users (equivalent to 56% of the UK population) needing to use the app in order to completely stop the pandemic spreading, although lower rates of use could still have beneficial impacts.^9^ Although much has been written about contact tracing apps in the media, academic research is only just beginning to explore public attitudes towards COVID‐19 policy, including contact tracing strategies and technologies, and much of it so far has taken the form of public opinion surveys.^14^, ^15^ Some surveys have suggested that a majority of smartphone users would use the app,^16^ but emerging evidence from early‐adopting countries like Singapore suggests that actual uptake might be considerably lower.^17^ There is, to our knowledge, as yet no published qualitative research on public attitudes towards COVID‐19 contact tracing apps. Qualitative research can provide an important supplement to large sample opinion polls involving an in‐depth exploration of the reasons and motivations behind individuals’ intentions towards use of contact tracing apps. If the UK’s contact tracing strategy is to be effective, government and health authorities will need to understand and respond quickly to any public concerns over the app. The aim of this paper is to explore attitudes towards digital contact tracing in a sample of the UK public. Specifically, we seek to explore people's knowledge of digital contact tracing, their views on contact tracing apps (specifically the extent to which they are favourable to them or not), and ultimately whether or not they planned to use a contact tracing app when one became available in the UK. These findings can feed into debates around how best to communicate to the public the potential role of digital contact tracing as a contributing intervention through which to reduce the future spread of COVID‐19.

## 2 METHODS

We conducted six online focus groups with 27UK‐based participants between 1st and 12th May 2020 during the UK’s COVID‐19 lockdown (39‐50 days after the official start of the UK lockdown).For context, as described above, at the time of data collection, the UK government had proposed a centralized approach using a NHSX‐developed app, due to be trialled from 4th May 2020. Participants were adults aged 18 years or over currently residing in the UK. All focus groups were conducted using the online conference platform Zoom. Online focus groups do have methodological benefits particularly as a cost‐effective method of eliciting public views from a geographically dispersed group of participants.^18^, ^19^

Focus groups were conducted as part of a larger, mixed‐methods longitudinal study tracking public attitudes to COVID‐19 and its associated policies in the UK, as well as the social and psychological impacts of such policies. Full ethical approval was granted by Swansea University's School of Management Research Ethics Committee. Focus group recruitment took place online through a combination of social media snowball sampling, online community and volunteer advertising sites and social media advertisements. For example, advertisements were placed in local community groups on social media (eg Facebook) and other community advertising sites (eg Gumtree), but did not target specific interest groups or inclusion criteria, other than focusing on local or regional interests. We did however take a purposive approach to sampling, attempting to include a range of ages, genders, racial/ethnic identities and occupational backgrounds (Table 1).

**TABLE 1:** Demographic details reported by participants

Characteristic	*N* (%)
Gender	
Female	13 (48)
Male	14 (52)
Age range	
18‐29	8 (30)
30‐39	7 (26)
40+‐49	8 (30)
Undisclosed	4 (14)
Ethnicity	
White—British	13 (48)
Other ethnicity	11 (41)
Undisclosed	3 (11)

Most participants were recruited from social media snowball sampling and online community and volunteer advertising sites, which likely accounts for the high number of participants in the 18‐49 age range and the lack of participants aged 50 and over (due to the fact the sites are more commonly used amongst younger age groups). Although participants were aware all research was online, adverts were placed in relevant community groups on social media and volunteering sites in a variety of countries, regions and cities across the UK, so as to recruit participants from across the UK The final sample included participants from all four UK countries (in order to protect confidentiality specific locations of participants are not provided). Paid‐for social media (Facebook) advertisements were taken out to specifically target potential participant aged 50 and over, but these failed to generate any sustained interest from potential participants.

Focus groups met for approximately one hour each. Most participants joined Zoom using both video and audio, although some were given the option, and preferred, to join with audio only. All focus groups were organized and moderated by SW (a medical social scientist). The topic guide for the focus groups was initially developed by the research team in virtual group meetings. Although the focus groups discussed topics related to the broader research project (eg experiences on social distancing, views on COVID‐19 testing and vaccinations), it also specifically included questions seeking to elicit participants views on COVID‐19 contact tracing apps (eg ‘do you think you will use the planned COVID‐19 app?’ and ‘what are your views on the planned COVID‐19 contact tracing app?’

### 2.1 Analysis

Data were analysed in accordance with a thematic approach^20^, ^21^ This is an iterative and abductive analytical process through which emergent themes from early focus groups were used to add to or refine questions and prompts during subsequent groups. Data were transcribed for coding, with all participant information being anonymized to protect confidentiality. SW and KD analysed the transcripts and developed and applied the thematic coding framework, moving from open through to more focused coding.^20^, ^21^ Themes were discussed and developed with CA and TT during virtual research group meetings. Negative case analysis was used to seek for information that did not fit emergent themes, and where this occurred, themes were modified accordingly.^22^ Data collection and analysis continued until theoretical saturation was reached.^21^ Data were analysed in NVivo (version 11.4.3, QRS).

Ethical restrictions related to participant confidentiality prohibit the authors from making the data set publicly available. During the consent process, participants were explicitly guaranteed that the data would only be seen my members of the study team. For any discussions about the data set please contact the corresponding author ( s.n.williams@swansea.ac.uk ).

## 3 RESULTS

Participants were split regarding whether or not they felt they would use a contact tracing app. While some stated they would be using the app, others argued the would not, and still others were unsure or undecided as to whether they were going to use it or not. No patterns emerged according to the demographics of the participants in terms of whether they felt they would use a contact tracing app or not.

Analysis revealed five main themes: (a) lack of information and misconceptions surrounding COVID‐19 contact tracing apps; (b) concerns over privacy; (c) concerns over stigma; (d) concerns over uptake; and (e) contact tracing as the ‘greater good’. These themes were found across the sample and the three groups; however, certain themes were more salient, and appeared to carry more weight in influencing their views as to whether they would use the app or not, in some groups compared to others. Many participants across all three groups either expressed a lack of knowledge of, or displayed misconceptions about, COVID‐19 contact tracing apps. Concerns over privacy and uptake were expressed across all three groups, but seemed to be particularly significant for those who said they would not use the app. Concerns over stigma were expressed by participants in the ‘would not use app’ and ‘undecided’ groups, but not in the ‘would use’ group. The view that contact tracing was for ‘the greater good’ was expressed by participants in the ‘would use’ and ‘undecided’ groups, but not in the ‘would not use’ group, and was particularly significant for those in the ‘would use’ group.

### 3.1 Lack of information and misconceptions surrounding COVID‐19 contact tracing apps

Many participants either expressed a lack of knowledge of contact tracing and contact tracing apps or appeared to have misconceptions over what the official UK government‐proposed app entails. Amongst those who had not heard of contact tracing apps, one of the reasons was because they had ‘*not been watching the news*’ (Participant 19, female, 20s), as they were ‘*sick and tired of hearing more of coronavirus*’ (Participant 20, female, 20s). Amongst those who had heard of them, one of the biggest misconception was that the app would entail some form of ‘mapping’ that would be visible to other smartphone users or the public in general:

I think they wanted to develop an app or something, so you would know how many Covid cases are in your area, so you know where not to go. So, if everyone got sent a test and managed in certain areas not go out until everyone has been tested, so you know where the virus is so you know who can leave their homes and who can’t (Participant 6, male, 20s)

### 3.2 Concerns over privacy

The most commonly stated concern was over data privacy and security. Participants expressed a reluctance to have their data accessed by government or health authorities. Participants associated contact tracing with increased surveillance by government and were concerned by what they perceived as ‘submitting’ their personal information:

Contact tracing seems quite Big Brotherly. I don’t think I am willing to submit all my data and all of my contacts for the government to scrutinise who I see regularly. I don’t think I will be willing to join the contact tracing apps (Participant 16, female, 30s)

Participants were also concerned that their data would be accessible to others outside of government and health authorities, including ‘third parties’ or ‘hackers’:

You don’t know who is running these apps, you don’t want third party accessing your information. This is the worrying thing about modern technology; you do not know who is accessing your health and things. (Participant 10, male, 40s)

Hackers could pose as health experts or something, there are so many scams that are going on. Scammers are supposedly selling. I am not in favour of this app personally. (Participant 7, male, 20s)

However, some of these concerns over privacy seemed to stem from the misunderstanding discussed in the previous section that app users would be able to identify specific COVID‐19 cases amongst their contacts or their vicinity:

Imagine walking around, and thinking “you’ve got it, you’ve got it” and you have got it [phone] in your hand … Imagine you were like, “oh I’ll go see my friends when we are all allowed,” and they were like “You have got corona it says it on my app” (Participant 19, female, 20s)

For those who stated they will not use the app, privacy was the most common reason stated for their decision. They were generally opposed to anyone, including government and health officials accessing their data under any circumstance. For those who said they would download the app, privacy concerns were mentioned, but were seen to be outweighed by the perceived benefits of the app for public health (as discussed below). Those who were undecided tended to argue that they would use the app provided they were sure that their data were not visible to other smartphone users or the general public they were given assurances over privacy and data protection:

I would get it if there were no privacy concerns and it was just for the government to track the spread of the virus … but I wouldn’t do it if there was a map to see. (Participant 18, female, 20s)

Those who were undecided also tended to weigh up their concerns over data privacy with their view that apps which require the disclosure of personal information are already commonplace:

I’m a bit wary with the app but I tend to download anything and sign my life away all the time … I think I would be alright with that kind of stuff …. I think everyone is already traced and they don’t even know. (Participant 16, female, 30s)

What do I have to hide? Nothing. Big Brother can watch me I really don’t worry about these things. But you never worry about it until something weird comes up and companies start trying to buy the data and do bad things with it. It’s all about ethics at that point. (Participant 26, Male 30s)

### 3.3 Concerns over stigma

Another commonly expressed concern was over the stigmatizing potential of the app. This concern was related to concerns over a lack of privacy and specifically the misconception that the app would enable people to use the app to identify others that have or have had COVID‐19:

I actually think that [the contact tracing app] is a terrifying concept… it’s like being branded with a. horrendous black mark. … I could look and be like my friend, my neighbour has got Covid. (Participant 17, male, 20s)

These participants feared that being able to identify others with COVID‐19 would lead to discrimination:

It could cause hate crime as well. Finding out “oh you know I got it from this person,” and finding out your neighbours have it, and “oh I will stay away from them.” (Participant 9, female, 30s)

It is just asking for more isolation and hysteria. … It would be like Snap Maps: “oh I want to go to Tesco, but oh no she’s there and she has got Covid”…. I think that would make me not want to get tested [for covid‐19]. I wouldn’t want to be on the “Snapmap of Shame.” Like, if you could see all these people that you know have corona, that could lead to bullying in some cases. (Participant 19, female, 20s)

### 3.4 Concerns over uptake

Another commonly voiced concern was over uptake of the app. A number of participants questioned whether enough people would use it in order for it to be an effective means of reducing the spread of the virus:

We have only reduced or flattened the curve because we have all collectively socially distanced … and if people were to download the app I think it would have to be done in a large group of people if not everybody for it to be effective … I don’t know how effective it would be in terms of transmission rates. I wouldn’t rush to download it. (Participant 17, male, 20s)

Amongst those who had heard of contact tracing, some drew comparisons between the UK and countries who had experienced the pandemic earlier and who had already made, in their view, effective use of contact tracing, such as Singapore. However, these participants tended to argue that the high level of adherence and enforcement in such countries would not be achievable in the UK which was perceived as less accepting of state interventionism and less collectivist:

The issue here is will society take it on board and actually do it. I think one of the reasons why places like China, South Korea, Singapore, those Asian countries can successfully manage those situations is because they have a relatively compliant society, people tend to work together or maybe it’s just because they are used to having their civil liberties curbed to a degree (Participant 12, male, 40s)

Another consideration was that a proportion of the population did not have smartphones, for example the homeless, elderly or socially or economically vulnerable in society. For some, such inequalities in access to the technology would reduce the effectiveness of using an app as part of a contact tracing strategy:

Some people don’t know how to download an app, some people don’t have a smartphone, some people may be left out, the vulnerable, and if they aren’t able to put in the data this might have an effect on other people. So how accurate and how up to date and how reliable the information could be would make a difference (Participant 1, male, 30s)

Amongst those who were undecided, there was a feeling that social norms or ‘pressure’ would lead them to overcome their reservations and use the app:

I’m torn. But if it gets to that point that its widely adopted, I probably would [use it]. Id cave to the pressure of like, “well everyone else is doing it.” But right now I feel very uncertain about it. (Participant 23, female 40s)

### 3.5 Contact tracing as ‘the greater good’

No participants were unequivocally positive about the contact tracing app. Ultimately, what distinguished those who were intended to use the app from those who did not intend on using it was their belief that it was the ‘right thing’ to do because it would be beneficial for the wider public health, and that this potential benefit outweighed their concerns:

If it is going to be for the greater good, I would do it, but it doesn’t fill me with joy. (Participant 17, male, 20s)

I would do it if it helps but it’s not something I want to do. (Participant 8, female, 40s)

In particular, the recognition of the severity and urgency of the pandemic was seen as a reason as a reason to use the app:

I would really support it, I know privacy is really important … anything that would help, it doesn’t make sense why people wouldn’t participate; people are dying all over the world, what’s more important at the moment, to try and stop this or people knowing what’s happened on your phone (Participant 16, female, 30s)

These participants implied that, because contact tracing in their view was necessary for slowing the spread of the virus, they had little agency over whether or not to use the app. Contact tracing was here characterized as a necessary evil (the ‘only way out’) or as an obligation (‘civic duty’):

The idea of tracking, with a Big Brother or something, I am totally opposed to it normally, but I can’t tell you how totally opposed normally. But based on what countries that have been successful have done, I feel like this might be the only way out of this. (Participant 21, female, 50s)

I don’t know, part of me thinks it is Big Brother, and I want to opt out completely, but part of me thinks it is my civic duty. I’m very, very torn. (Participant 27, Male 30s)

## 4 DISCUSSION

Our findings suggest five interrelated factors which shaped our participants’ perceptions of COVID‐19 contact tracing apps in the UK; concerns over uptake, stigma and privacy; a lack of information or misinformation about the nature and function of the app; and the perception of contact tracing as the ‘greater good’. Overall, our group of participants were torn as to whether they felt contact tracing apps were useful as a public health intervention and as to whether they would use them or not. Ultimately, the latter rests on the former, since if enough people feel that a contact tracing app is not going to effective because too few others are not going to use it (and use this as a reason to not use it themselves) then their beliefs may contribute to a self‐fulfilling prophesy at the population level. Concerns over uptake were framed in terms of social inequalities and cultural norms around state interventionism. It was argued that because certain social groups do not have access to smartphones, and because the UK public would not be willing to accept contact tracing in general as a strategy, it would not be as effective as it had been in countries like Singapore or South Korea. Participants suggested that social ‘pressure’ might lead them to use the app, suggesting that uptake is tied to social norms around the potential popularity and thus effectiveness of the app. In turn, social norms over the popularity and eventual uptake of the app are interrelated with additional concerns over privacy and stigma, as well as to perceptions of social obligation (‘civic duty’).Concerns over privacy were often framed in terms of a general lack of trust or faith in government and its handling of the pandemic thus far, and their opposition to what was perceived as an unnecessary form of government control and the role of technology in the rise of a surveillance society.^23^, ^24^, ^25^

A major theme was the perceived lack of knowledge and the existence of misconceptions around the app. One of the common misconceptions was that the app would provide identifiable information, and users themselves would be able to specifically identify others with COVID‐19 (or be identified themselves). These concerns were expressed by participants across all three groups, but, as discussed below, were more salient in some groups over others, in relation to influencing their view on whether they will likely use the app or not. Concerns over stigma stemmed from a fundamental misconception that the app could enable its users to identify COVID‐19 cases amongst their contacts or even synchronously map cases near them. It is worth noting that, despite these misconceptions, such findings imply that COVID‐19 may be, for some, becoming a stigmatized disease. Future research will explore the potential stigmatization of COVID‐19 sufferers in more depth and over time. The main implication of relevance to the present study is that the public may not be adequately informed as to what contact tracing apps entail. Improved communication regarding the purpose and nature of contact tracing apps may lead to increased use of the app.

Decision making was heavily influenced by moral reasoning. Those who said they would not download the app were motivated primarily by their moral opposition to the use of such technologies as an infringement of individual civil liberties. This can be seen as an example of the liberal objection to public health intervention,^26^ as seen previously in debates over smoking bans,^27^ electronic cigarettes^28^ and sugar taxes.^29^ By contrast, amongst those who were intending on using the app, decision making was driven by a more utilitarian evaluation of the relative costs and benefits.^30^ Many shared the same concerns as those who did not intend to use the app, but deemed that the potential life lost from COVID‐19 outweighed any privacy infringements. In a sense, their construction of the app as being for ‘the greater good’ serves as rejoinder, based on Mill's harm principle,^31^ to the liberal objection that using the app is a personal choice (since by not using it, one can, indirectly harm others by contributing to the spread of the virus).In some cases, reluctant willingness to use the app was framed in altruistic terms, as a personal sacrifice of one's privacy, whereas in other cases it was framed more in terms of enlightened egoism,^32^ that is, as a means of protecting the vulnerable while also getting themselves ‘out of this’ pandemic. For those who stated they would use the app, the decision was arguably one high in ‘moral intensity’, because of the temporal immediacy of the pandemic (its rapid spread), the magnitude of its consequences (its high death rates) and its concentration of effect (ie severe impacts (serious illness and death) amongst a relatively small number of people being greater than modest impacts (privacy risks) amongst a much larger number of people (total app users)).^33^ Finally, those who were undecided were generally still in the process of a utilitarian evaluation of the perceived costs and benefits of the contact tracing app. In order to achieve initial and ongoing use of contact tracing apps that are high enough to be effective,^9^ it will be important to obtain the support of those who are as yet undecided as to whether such apps are an effective and ethical public health intervention.

It is worth mentioning that a novelty of the methodology of this study is the fact that online focus groups were rendered necessary due to the fact that in‐person focus groups were not permitted as a result of the UK social distancing policy. As such, this provides an early example of the potential feasibility of conducting qualitative research during a pandemic. It is likely that currently, and for at least the immediate future, much qualitative research will need to be conducted via platforms such as Zoom. Future publications, building on our longitudinal design, will explore in greater depth the implications of virtual data collection during the pandemic, but a couple of early reflections warrant discussion here. Firstly, participant recruitment might have been impacted by the fact that far more social interaction was occurring online due to the pandemic. For instance, some potential participants might have been deterred from participating because they were already engaging in numerous videoconferences and were reluctant to add more to their schedules (a number of participants in our study mentioned feeling as though they were spending excessive time online (and suffering from so‐called ‘Zoom fatigue’). Secondly, although online focus groups were used in this particular study as a means for data collection, discussions revealed how for some participants, particularly those who lived alone, the focus groups themselves served as a source of social contact at a time when many in society were feeling isolated.^4^ Participants reflections of the experience of taking part in the focus groups themselves will be discussed in more detail in future publications.

### 4.1 Limitations

A limitation of this study is its sample size, which is small in relation to large quantitative surveys,^16^ and thus does not permit our findings to be readily generalized to the UK population as a whole. This, however, is a limitation inherent to all qualitative research and not specifically to the current study. The main contribution of this study is its ability to shed light on the underlying reasons and beliefs that account for people's views on the app and which ultimately shape their decision of whether or not to use it. This study is also limited insofar as it did not recruit participants from ‘clinically extremely vulnerable’ categories.^34^ The terms ‘clinically vulnerable and clinically extremely vulnerable’ are official terms used by the UK’s NHS and denote those groups deemed at ‘moderate risk’ and ‘high risk’, respectively, with high risk groups including those who have a serious heart condition or who are undergoing certain treatments for cancer.^34^ Additionally, the study did not include any individuals aged 70 or older (also considered ‘clinically vulnerable’, although this was because we did not receive any responses from this age group, despite noting in our recruitment material that applications from those considered clinically vulnerable were particularly encouraged. It is worth noting that using the term ‘vulnerable’ in our recruitment material may have dissuaded some participants from responding to our recruitment material, since, as has been argued, the term may be considered in some cases as derogatory or stigmatizing.^35^ Future research will look to specifically explore the attitudes of those classed as clinically extremely vulnerable(eg with specific health conditions) and clinically vulnerable (eg over 70 years old) on COVID‐19‐related policy.

## 5 CONCLUSIONS

Overall, findings suggest that many people are torn over whether digital contact tracing is a good idea or not, and concerns, particularly over privacy and effectiveness play a significant role in their decision making. Several of our participants may have been insufficiently informed about the specific details of what digital contact tracing entails. Interestingly, our findings suggest that some people may be actively avoiding news coverage on coronavirus (as a result of over‐exposure to COVID‐19 coverage and/or as a coping or avoidance strategy). Indeed, limiting watching news coverage on COVID‐19 has been recommended as a means to protect mental health during the pandemic.^36^ As such, authorities must explore a range of methods and media to communicate the purpose and nature of contact tracing apps, including but not limited to, social media ads, postal information, text messaging and other emergency alert systems.

Public health authorities need to explore ways to better communicate or emphasize that the app cannot enable the user to identify which of their contacts has reported COVID‐19 symptoms or tested positive, and that confidentiality is protected (since, at time of data collection this was not clear to most participants).Due to the longitudinal nature of the research, future papers will explore participants’ actual use or non‐use of the app. In order to for uptake to be high, normative beliefs will need to reflect the potential effectiveness and popularity of the app. In this way, public health messages could be similar to those employed in the effort to encourage widespread adherence to social distancing guidelines, including campaigns around collective responsibility and the ‘greater good’. In engaging with the public, government and health agencies should also take into consider the range of ethical orientations through which people are forming their views, something that may also prove useful in understanding public attitudes to other COVID‐19 policies such as testing, self‐isolation and particularly vaccines. The COVID‐19 pandemic is a fundamentally global public health issue. As such, policymakers need to communicate to, and engage with, publics in ways which critically explore the interdependence of individual and population risk‐taking and how self and how, in the context of global public health, self‐interest and altruism can often converge.^37^

## CONFLICT OF INTEREST

Armitage is supported by NIHR Manchester Biomedical Research Centre and NIHR Greater Manchester Patient Safety Translational Research Centre. Tampe is an independent consultant and currently consults for the World Health Organization. The authors have no other relationships or activities that could appear to have influenced the submitted work.

## Data Availability

Data are available upon reasonable request. Ethical restrictions related to participant confidentiality prohibit the authors from making the data set publicly available. During the consent process, participants were explicitly guaranteed that the data would only be seen by members of the study team. For any discussions about the data set, please contact the corresponding author: SNW ( s.n.williams@swansea.ac.uk ).
